# Immunomodulatory constituents of human breast milk and immunity from bronchiolitis

**DOI:** 10.1186/s13052-017-0326-3

**Published:** 2017-01-14

**Authors:** Chunyu Li, Yanbo Liu, Yanfang Jiang, Naijun Xu, Jie Lei

**Affiliations:** 1 Department of Pediatrics, the First Hospital of Jilin University, No.3302 Jilin Road, Changchun, 130021 China; 2 Key Laboratory for Zoonosis Research, Ministry of Education, the First Hospital of Jilin University, Changchun, 130021 China

**Keywords:** Immunomodulatory, Breast milk, Bronchiolitis, Children

## Abstract

**Background:**

The mother’s immune status can be achieved by genetic and breastfeeding impact descendants of the immune system. The study aimed to determine whether a mother’s immune status and breastfeeding practices were related to development of bronchiolitis in her infant.

**Methods:**

The frequency of T, B and natural kill (NK) cells in patients’ blood and their mothers’ breast milk was determined using flow cytometry. The concentrations of serum and breast milk IgG and IgD in individual patients and healthy control were determined by enzyme-linked immunosorbent assay (ELISA). The relationships between immunocytes, immunoglobulin and respiratory score (RS) were analyzed by Spearman’s rank correlation test.

**Results:**

The mothers of bronchiolitis patients had lower IgG concentrations in their breast milk when compared to the mothers of healthy children. There was no significant difference in the frequency of T cells, B cells, and NK cells in samples of breast milk. However, significant decreases of CD3+, CD8+ T cells, as well as significant increases of CD4+ T cells and CD19+ B cells were found in the serum of bronchiolitis infants. There were positive correlation relationships between RS and CD3+, CD4+ T cells, IgG and IgD concentrations.

**Conclusion:**

Our data suggested that the mothers of bronchiolitis patients had lower IgG concentration in their breast milk. The breast milk IgG might be absorbed by the breastfeeding infants, which could play important role in resistance of bronchiolitis.

## Background

Bronchiolitis is one of a common respiratory disease that predominantly happened in children aged less than 2 years old [1], which is often caused by respiratory syncytial virus (RSV) and presents with clinical symptoms of wheezing, tachypnea and cough [2]. The infected patients experiences several days of congestive symptoms before they resolve spontaneously. Substantial evidence has indicated that compared to infants with normal pulmonary function, infants with underlying airway hyper-responsiveness are likely to display more extensive clinical symptoms of a RSV infection [3]. Though a previous study has suggested that children who underwent wheezing might have high risk of developing asthma and allergic disorders [4], the evidence for a link between atopic asthma and bronchiolitis is still difficult to interpret; and thus whether any relationship exists between these two disorders remains uncertain [5]. However, it is generally accepted that asthma is often associated with dysregulation of the immune system, which makes asthma patients particularly susceptible to infections.

Patients with asthma have increased number of CD4 + T lymphocytes, decreased CD8+ T cells as well as higher ratios of CD4+/CD8+ [6]. As part of the innate immune system, natural killer (NK) cells represent the first line of defense against infection, and are capable of directly killing target cells and interacting with both antigen-presenting cells and T cells [7]. Though NK cells have received increased attention in recent years, only few studies have investigated their numbers and functions in bronchiolitis patients.

For breastfeeding babies, the composition of breast milk significantly affects their immune function and development [8]. Breast feeding provides an infant with several soluble factors directly involved in their mucosal defenses [9]. It appears sensible to avoid allergy, sensitization and even allergy-related diseases [10]. The relationship between breast feeding and bronchiolitis has been discussed previously. However, the evidence on such relationship still remains conflicting. For example, Dixon et al. [11] examined the IL-8 levels in nasopharyngeal aspirates obtained from children with acute bronchiolitis, and found that children who were being breast fed had lower levels of IL-8 and lower numbers of inflammatory cells in their aspirates when compared to children who were not being breast fed. Such findings suggest the beneficial effects of breast feeding on reducing the incidence of infant bronchiolitis. However, Duncan et al. [12] reported that the data did not prove any association between breast feeding and bronchial asthma and other allergic diseases. Therefore, whether breast feeding children with bronchiolitis helps to protect against its symptoms requires more evidence to clarify.

We performed this study based on the data of 20 infants and their mothers with bronchiolitis to determine whether the mother’s immune status and breastfeeding practices were related to development of bronchiolitis.

## Methods

### Patients and controls

From October 2013 to May 2014, a total of 20 infants (11 males and 9 females, mean age 8 months; age range, 3–15 months) with bronchiolitis were sequentially recruited to participate in this study conducted at the inpatient facility of the First Hospital of Jilin University. A separate group of 11 gender- age- and ethnicity-matched healthy control subjects with no previous history of a respiratory tract infection were recruited during the same time period. The recruitment of controls helped to minimize differences in breast milk constituents. Each case of bronchiolitis was diagnosed using established international criteria (14). Each infant included in the study received breast feeding only. Bronchiolitis patients who had received post-natal treatment with corticosteroids or intravenous immunoglobulin, or had another disease such as congenital heart disease, anemia, malnutrition, vitamin D deficiency or bacterial diarrhea, were excluded from the study. Samples of breast milk were also obtained from a separate group of 11 postpartum age-matched women with a healthy breast feeding child.

The protocol for this study was approved by the Ethical Committee of the First Hospital of Jilin University, and each mother provided a signed written informed consent prior to enrollment.

The demographic (age and gender) and clinical characteristics of each participant were recorded by physician, and were shown in Table 1. The blood samples were collected from each infant on the second day of hospital admission, and 10 mL breast milk from each mother was collected.

**Table 1:** Clinical and laboratory characteristics of the bronchiolitis patients and healthy control subjects

	Bronchiolitis group (*n =* 20)	Healthy group (*n =* 11)
Age (months and range)	8 (3–15)	9 (4–16)
Male/female	11/9	6/5
Onset time (months)	13.5 (3–24)	0
Respiratory score (RS)	2 (1–3)	0
WBC count (× 10^9^/L)	2.45 (1.2-4.7)	6.6 (4.9-8.3)
Lymphocytes (× 10^9^/L)	2.86 (1.77-3.75)	1.56 (1.25-2.91)

*WBC* white blood cell count; Normal values, WBC, 3.5-9.5 × 10^9^/L; lymphocytes, 1.1-3.2 × 10^9^/L

### Collection of peripheral blood mononuclear cells (PBMCs)

To minimize intra-feeding variations and limit diurnal variations during the morning, each mother completed a full expression of milk from one breast at least 2 h following the last feeding from that breast. These breast milk samples were maintained at 4 °C and processed within 4 h after collection. Each sample of breast milk was centrifuged at 1500 g for 5 min, and the acellular fraction (lactoserum and lipid fraction) layer was removed. The fat layer was poured off and the supernatant fraction was precipitated with physiological saline. The precipitate was pelleted by centrifugation at 1500 g for 5 min; after which, it was washed two times and then re-pelleted. The pelleted cells were resuspended in culture medium (RPMI-1640) and adjusted to a concentration of 1 × 10^6^ cells/L. PBMCs were obtained by standard Histopaque density centrifugation, and the BMCs and PBMCs were analyzed by flow cytometry.

### Flow cytometry analysis

Duplicate samples of human PBMCs (10^6^/sample) were stained with the following regents for 30 min at room temperature: fluorescein isothiocyanate conjugated anti-CD 4 (FITC-anti-CD4), phycoerythrin conjugated anti-CD8 (PE-anti-CD8), peridinin-chlorophyll-protein conjugated anti-CD3 mAb (PerC*P-*anti-CD3; Clone SK7/SK1/SK3; BD Tritest; San Jose, CA, USA), PE-anti-CD19, FITC-anti-CD138, PE-anti-IgD, FITC-anti-CD3, and PE-anti-CD16 + CD56+ (BD Bioscience; San Jose, CA, USA). Samples stained with FE-anti-IgG, FE-anti-IgG1, FE-IgG2a, and FITC-anti-IgG (BD Bioscience) were used as isotype controls. After staining, the cells were washed with PBS and analyzed using a FACSAria II flow cytometer (BD BioSciences; Franklin Lakes, NJ, USA). A minimum of 50,000 events per sample were analyzed using FlowJo software (v5.7.2) (FlowJo LLC; Ashland, OR, USA) [13].

### Enzyme-linked immunosorbent assay (ELISA)

Concentrations of IgG and IgD in serum samples obtained from individual patients and healthy control subjects were determined using human IgG ELISA kits and human IgD ELISA kits, respectively, according to the manufacturer’s instructions (Cusabio; Wuhan, China). Briefly, 1:4 dilutions of sera were analyzed by ELISA, and the concentrations of IgG and IgD were calculated based on a standard curve established using manufacturer-provided samples of recombinant IgG and IgD. The lower limits for IgG and IgD detection were 0.487 ug/mL and 0.024 ng/mL, respectively.

### Respiratory score

Respiratory score (RS) [14] of each patient’s was calculated using data from four different physiologic parameters: respiratory rate, retractions, dyspnea, and auscultation. Twenty patients with bronchiolitis and 11 healthy control subjects were independently assessed.

### Statistical analysis

All data were analyzed using IBM SPSS Statistics for Windows, Version 19.0. Armonk, NY: IBM Corp. Differences between groups were analyzed using the Mann–Whitney U nonparametric test and relationships between variables were evaluated using Spearman’s rank correlation test. Data were expressed as either the median value and range or individual mean values. Two-sided *P*-values < 0.05 were considered statistically significant.

## Results

### Relationship between breast milk lymphocytes and bronchiolitis

Flow cytometric analyses of breast milk samples showed no differences in the frequencies of T cells, B cells or NK cells in the breast milk fed to bronchiolitis patients and healthy children (data not shown). The median number of CD4/CD8 (1.23 ± 0.69) and the median number of NK (9.36 ± 6.83) cells in breast milk is fewer than in serum (Data not shown).

### Relationship between serum lymphocytes and bronchiolitis

**Fig. 1:**
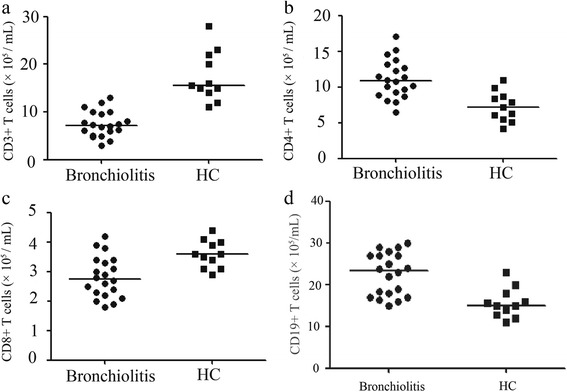
T lymphocyte subsets in bronchiolitis patients and healthy control subjects (**a**), (**b**), (**c**) and (**d**) show data regarding the numbers of CD3+ T cells, CD4+ T cells, CD8+ T cells and CD19+ B cells in bronchiolitis patients and healthy controls. Horizontal lines show the median value

Changes in lymphocyte subsets in samples of peripheral blood and breast milk obtained from the bronchiolitis patients were examined by the flow cytometry. We found fewer numbers of CD3+ T cells and CD8+ T cells (*P* < 0.001, *P* = 0.0014; Fig. 1a and c), but higher numbers of CD4+ T cells (Fig. 1b, *P* = 0.0003) in bronchiolitis patients compared to healthy control subjects. Additionally, the numbers of CD19+ B cells (*P* = 0.0056, Fig. 1d) in bronchiolitis patients were also much more than that of healthy control subjects. However, bronchiolitis patients and healthy subjects had similar numbers of NK cells (data not shown).

### Results of ELISA analyses of blood serum and breast milk

**Fig. 2:**
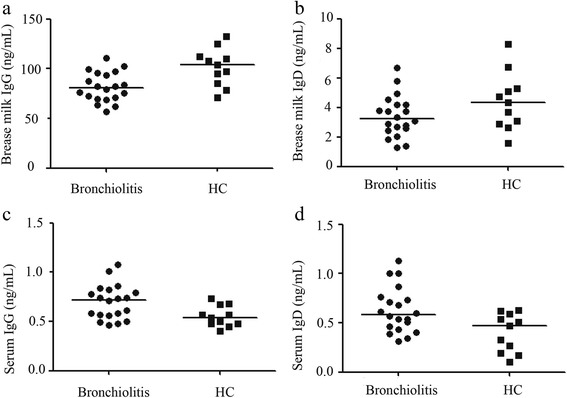
ELISA analyses of immune globulin in breast milk and blood serum (**a**) and (**b**): summarized data showing the numbers of IgG and IgD B cells in breast milk samples obtained from the mothers of bronchiolitis patients and healthy control subjects. Data are expressed as the mean values of individual samples; the horizontal lines represent median values. (**c**) and (**d**): Summarized data showing concentrations of IgG and IgD in the blood serum of bronchiolitis patients and healthy control subjects. Data are expressed as the mean value of individual samples and horizontal lines represent the median value

The mothers of bronchiolitis patients had lower IgG concentration in their breast milk when compared to the mothers of healthy children (*P* = 0.025; Fig. 2a), but blood samples obtained from bronchiolitis patients had more IgG concentration compared to samples obtained from healthy control subjects (*P* = 0.0187; Fig. 2c). Besides, blood samples obtained from bronchiolitis patients contained higher IgD level compared to those obtained from control subjects (*P* = 0.01; Fig. 2d). However, there was no significant difference in the IgD concentration detected in the breast milk samples obtained from the mothers of bronchiolitis patients and the mothers of healthy children (*P* = 0.1087; Fig. 2b).

### The relationship between the immunocytes, immunoglobulin and RS

**Fig. 3:**
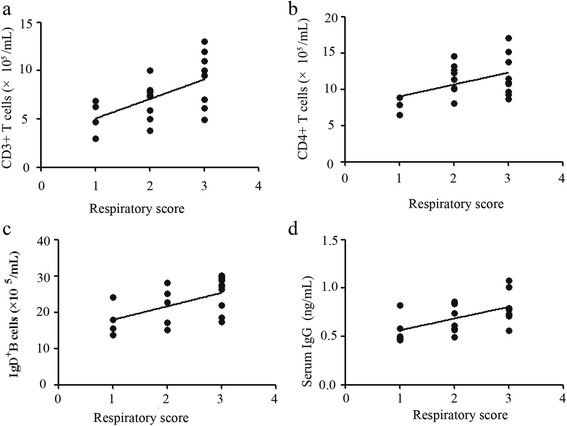
The relationship between the respiratory scores and number of immunocytes, immunoglobulins (**a**) and (**b**): the numbers of CD3+ and CD4+ T cells were positively associated with respiratory scores (RS). (**c**) and (**d**): the numbers of IgD+ B cells and the concentration of IgG were positively associated with respiratory scores (RS)

To better understand the importance of different T and B cell subsets, we analyzed potential relationships of these subsets with the values obtained for various clinical parameters in bronchiolitis patients. We found that the numbers of CD3+ and CD4+ T cells, as well as the concentrations of IgD+ B cells and IgG, were positively correlated with RS in bronchiolitis patients (Figs. 3a-d). However, no significant associations were observed between the RS and subsets of T cells and NK cells in samples of breast milk obtained from the mothers of bronchiolitis patients (data not shown).

## Discussion

Immunomodulatory constituents of human breast milk may affect the patient’s immune function. Children who are breasted have immunological advantage when compared with those who were fed with formula [15]. In this study, we analyzed the immunomodulatory constituents of human milk in response to bronchiolitis. Results showed that the number of breast milk IgG was fewer in mothers of bronchiolitis patients when compared to the mothers of healthy children, but the concentrations of serum IgG in patients with bronchiolitis were markedly increased. However, there were no significant differences of total T cells, B cells as well as NK cells between the two breast milk samples. For children serum immunocytes, the serum CD3+ and CD8+ T cells were significantly reduced but the serum CD19+, IgD B cells were significantly increased in patients with bronchiolitis.

T cells can damage tissue either directly by their cytolytic activity or indirectly by secreting proinflammatory mediators capable of recruiting and activating other types of immune cells [16]. In case of upper respiratory tract infection, CD4+ T cells assist in driving the B cell-dependent autoantibody response and modulating the activation and elimination of CD8+ T cells [17]. Our data showed that the mothers of bronchiolitis patients had lower IgG level in their breast milk when compared to the mothers of healthy children. Infants with bronchiolitis had fewer numbers of CD3+ T cells and CD8+ T cells, more number of CD4+ T cells, and higher CD4+/CD8+ ratios in their blood when compared with healthy control subjects. These findings are similar to those previously reported for asthma patients [18]. Respiratory scores consisting of a set of physical signs and clinical symptoms are usually used to estimate the severity of infant bronchiolitis [19]. We found that the RS was significantly and positively correlated with the levels of IgG and IgD, and the values for serum parameters in bronchiolitis patients. All these results suggest that bronchitis patients are in an immune disorder.

As the most important effector cells in humoral immunity, activated B cells are capable of recognizing an antigen, and then developing into plasma cells which secrete immunoglobulins. Later, these plasma cells are converted to memory cells. In our study, patients showed increased level of CD19+ B cells and IgD concentrations in their serum. The increased CD19+ B cells indicates that lymphocytes have participated in a humoral immune response [20], and B lymphocytes play important roles in both the synthesis and secretion of IgE. Additionally, high level of IgD was found in blood samples from bronchiolitis patients compared to samples from healthy subjects. The mothers of bronchiolitis patients had low IgG level in their breast milk when compared to the mothers of healthy children. However, concentrations of breast milk from mothers of patients and healthy individuals did not seem to be different. We assumed from these results that immunomodulatory constituents of human breast milk might affect the patient’s immune function.

In the innate immune system, NK cells are the first line of defense against infection, and are activated by cytokines [21]. Activated NK cells directly kill their target cells by releasing cytolytic granules which form pores in the membranes of target cells [22]. After killing their target cells, NK cells secrete cytokines that modulate the subsequent development of adaptive immunity. These functions of NK cells are regulated by the relative numbers of activating and inhibitory signals received by receptors on the NK cell surface [23]. Additionally, the cell recognition and killing capabilities of NK cells are finely regulated by the relative activities of multiple receptors with either activating or inhibitory functions [24]. In our present study, we found no significant difference between the numbers of NK cells in bronchiolitis patients and healthy control subjects.

Breast milk contains large numbers of T cells and somewhat fewer numbers of B cells and NK cells. We found no significant difference in the numbers of T cells, B cells or NK cells in the samples of breast milk obtained from mothers who had a child with bronchiolitis vs. mothers with a healthy child. T cells play important roles in the immune response and immune regulation, and while CD4+ T cells often promote humoral and cellular immune responses; whereas, CD8+ T cells can inhibit immune responses. The mean CD4+ / CD8+ cell ratio in blood is > 1.0, indicating that CD4+ T cells have numerical advantage. A previous study has confirmed that CD4+ T cells in breast milk has similar function to memory T cells [25], and this is consistent with changes seen in blood T cells. Macrophages play a defensive role in anti-infection immune reactions via rapidly initiating secondary immune responses to eliminate the invasive pathogen. Human breast milk is uniquely suited for consumption by infants, as proven by its high nutritional value and large array of non-nutritive but bioactive factors which promote survival and healthy development [26]. The exclusive use of human milk is a food source for infants during the first 6 months of life, and its continued use through ages 1–2 years is currently recognized as the normative standard for infant feeding [27, 28]. A study conducted by Chantry et al. [29] in the USA examined the effects of exclusive breast-feeding for 4 and 6 months on the incidence of pneumonia and otitis media in children, after adjusting for confounding factors, the results showed that the 4-month breast-feeding increased the risk of pneumonia and otitis media in children than that of 6-month breast-feeding. Additionally, a recent review of results from several individual studies indicated that breast feeding may enhance an infant’s long-term protection against certain infections, e.g., gastroenteritis, respiratory tract infections, skin infections, urinary tract infections, and severe complications resulting from measles [30]. Breast milk is not only a rich source of nutrients such as unsaturated fatty acids, lactose, vitamins, high-quality proteins, and taurine, but also helps to promote the development of infant’s brain. Additionally, breast milk contains molecules which are believed to regulate the development and maturation of immune cells (e.g., CD4+ T cells) and production of cytokines [31], to reduce IgG. Therefore, the significant decrease in the IgG concentration in breast milk in response to bronchiolitis suggests a specific immunological pathway against bronchiolitis.

## Conclusion

We conclude from these results that the decreased level of IgG in breast milk of mothers of bronchiolitis infants might associated with the immunological response to bronchiolitis. The breast milk IgG might be absorbed by the breastfeeding infants, which could play important role in resistance of bronchiolitis.
